# Volar Distal Radioulnar Joint Dislocation With Ulnar Styloid Fracture

**DOI:** 10.7759/cureus.109018

**Published:** 2026-05-17

**Authors:** Rashwan Elmeligy

**Affiliations:** 1 Orthopedics and Traumatology, Al Qurayyat General Hospital, Al Qurayyat, SAU

**Keywords:** dislocation of the distal radioulnar joint, distal radioulnar joint dislocation with ulnar styloid fracture, volar distal radioulnar joint dislocation, volar wrist dislocation, wrist dislocation

## Abstract

Volar dislocation of the distal radioulnar joint (DRUJ) is an uncommon traumatic injury that may be overlooked due to subtle clinical findings and difficulty obtaining proper radiographs in the acute setting. The mechanism typically involves a strong pronation force applied to a fixed wrist, leading to disruption of the stabilizing soft tissue structures, particularly the triangular fibrocartilage complex (TFCC). Although standard wrist radiographs are recommended initially, computed tomography (CT) is often required when positioning is inadequate or the diagnosis remains uncertain.

We report a rare case of volar DRUJ dislocation associated with an ulnar styloid fracture in a 36‑year‑old man following a fall on an outstretched hand. Closed reduction was successfully performed in the emergency department, followed by immobilization and structured follow‑up. The patient achieved full functional recovery by 11 weeks. This case highlights the importance of early suspicion, appropriate imaging, and timely management to prevent long‑term complications.

## Introduction

Volar dislocation of the distal radioulnar joint (DRUJ) is a rare injury, with most DRUJ dislocations occurring dorsally [1]. The injury typically results from a forceful pronation mechanism that disrupts the triangular fibrocartilage complex (TFCC) and other stabilizing structures [2]. When pain and swelling limit proper radiographic positioning, the diagnosis may be easily missed.

Fewer than 30 cases of isolated volar DRUJ dislocation have been reported, and only a small subset involved an associated ulnar styloid fracture [3]. Recent publications between 2022 and 2024 have expanded the understanding of this injury pattern and emphasized the diagnostic challenges and the importance of computed tomography (CT) imaging when radiographs are inconclusive [4-6].

## Case presentation

A 36‑year‑old man presented to the emergency department after falling on an outstretched hand while the forearm was in a pronated position. He reported severe wrist pain and significant limitation of both supination and pronation.

Clinical examination revealed moderate swelling of the left wrist with the forearm fixed in pronation. Rotational movements were markedly restricted due to pain. Distal perfusion was intact, with a palpable radial pulse and normal capillary refill. No sensory or motor deficits were identified.

**Figure 1:**
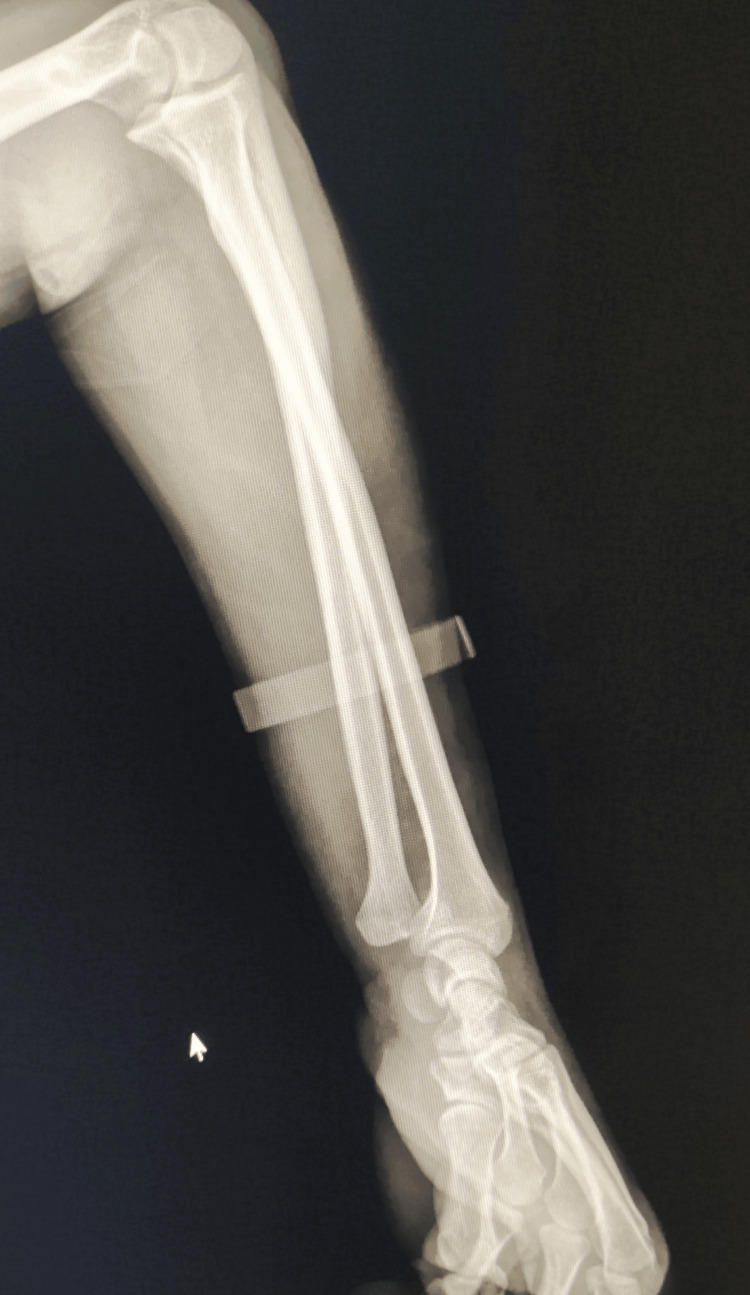
Lateral wrist X-ray Oblique lateral wrist radiograph with inadequate visualization of the distal radioulnar joint due to patient discomfort.

**Figure 2:**
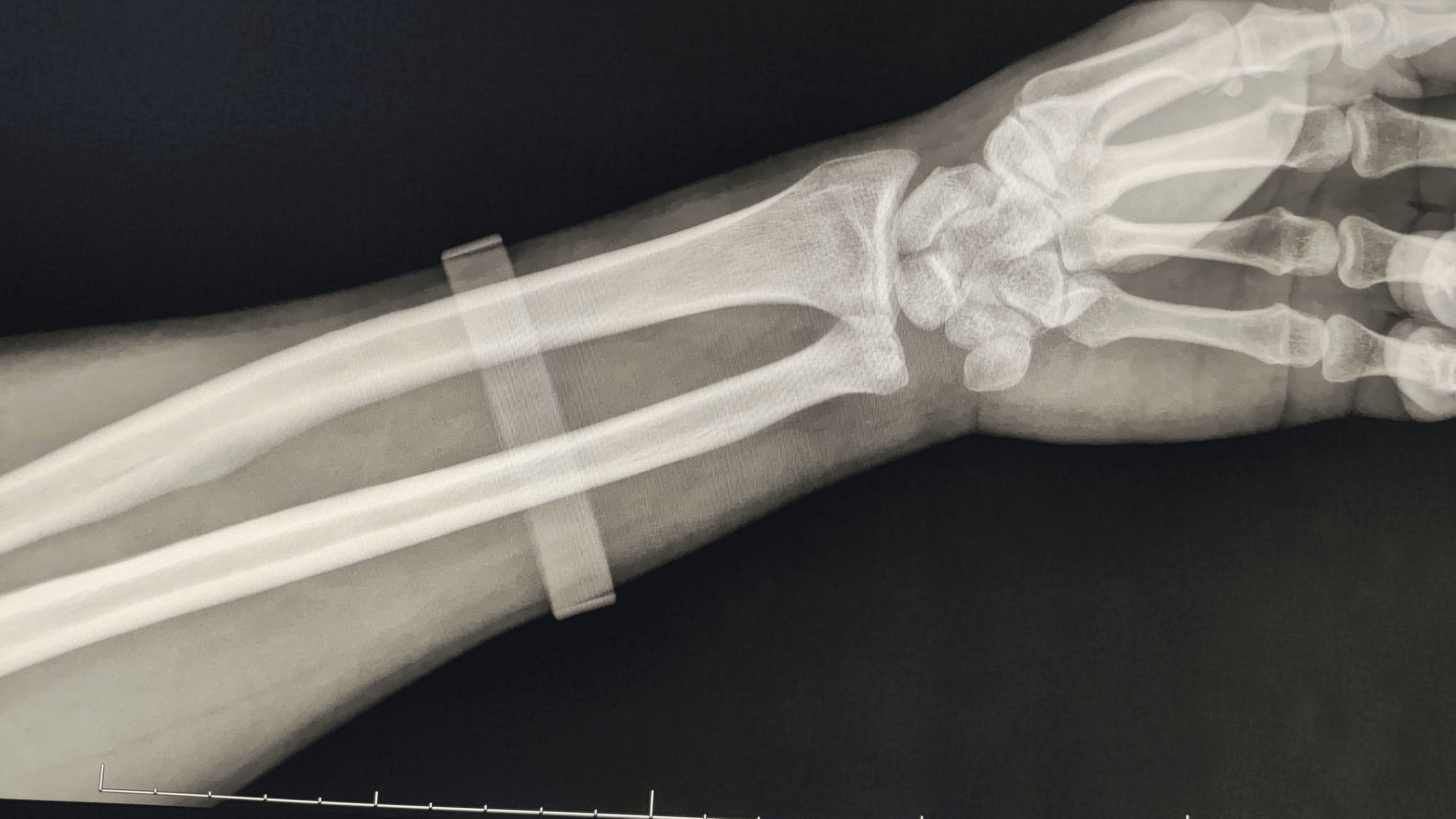
Anteroposterior wrist X-ray Anteroposterior wrist radiograph showing suboptimal positioning and poor visualization of the ulnar styloid.

Initial wrist radiographs were obtained. The lateral view (Figure 1) was oblique due to patient discomfort, limiting the assessment of the DRUJ. The anteroposterior view (Figure 2) also demonstrated poor positioning, making the visualization of the ulnar styloid difficult.

**Figure 3:**
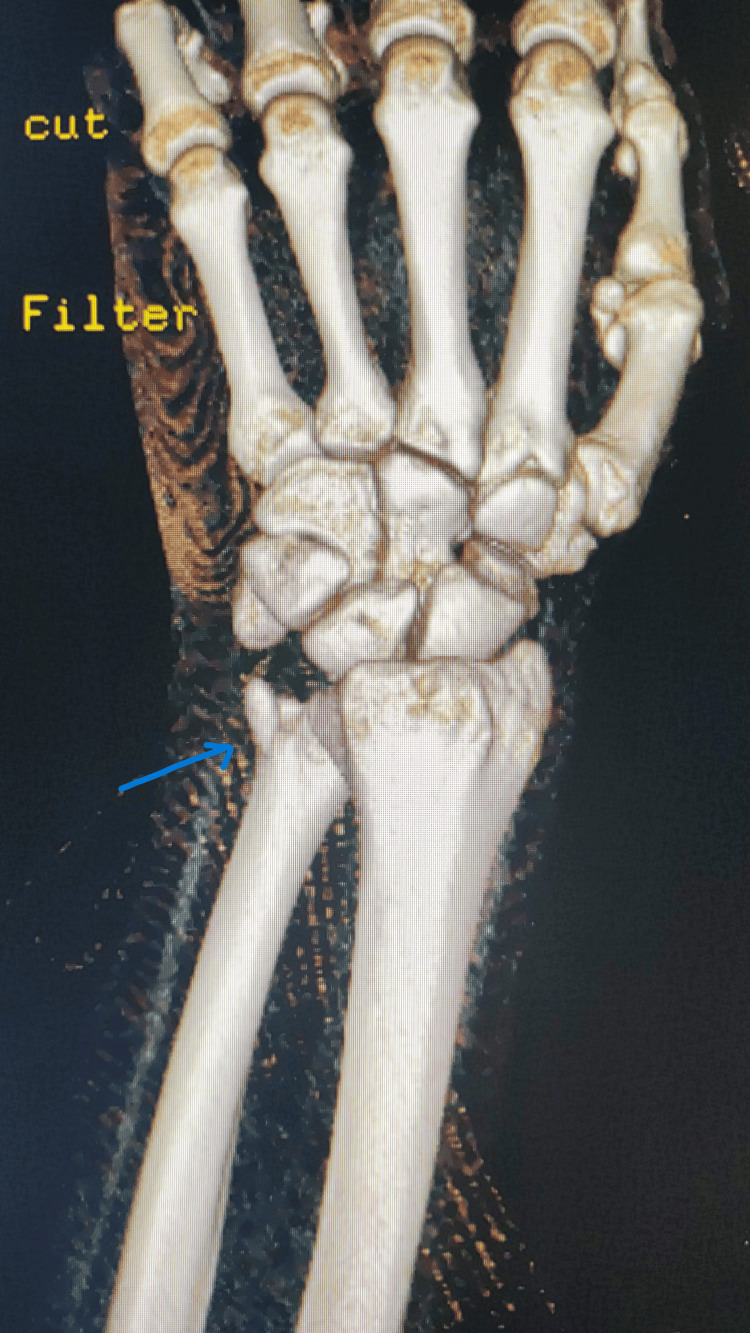
3D computed tomography Computed tomography 3D reconstruction demonstrating volar dislocation of the distal radioulnar joint. Blue arrow pointing to the ulnar styloid process. Figure created using Philips IntelliSpace PACS (formerly known as iSite) (Amsterdam, Netherlands).

**Figure 4:**
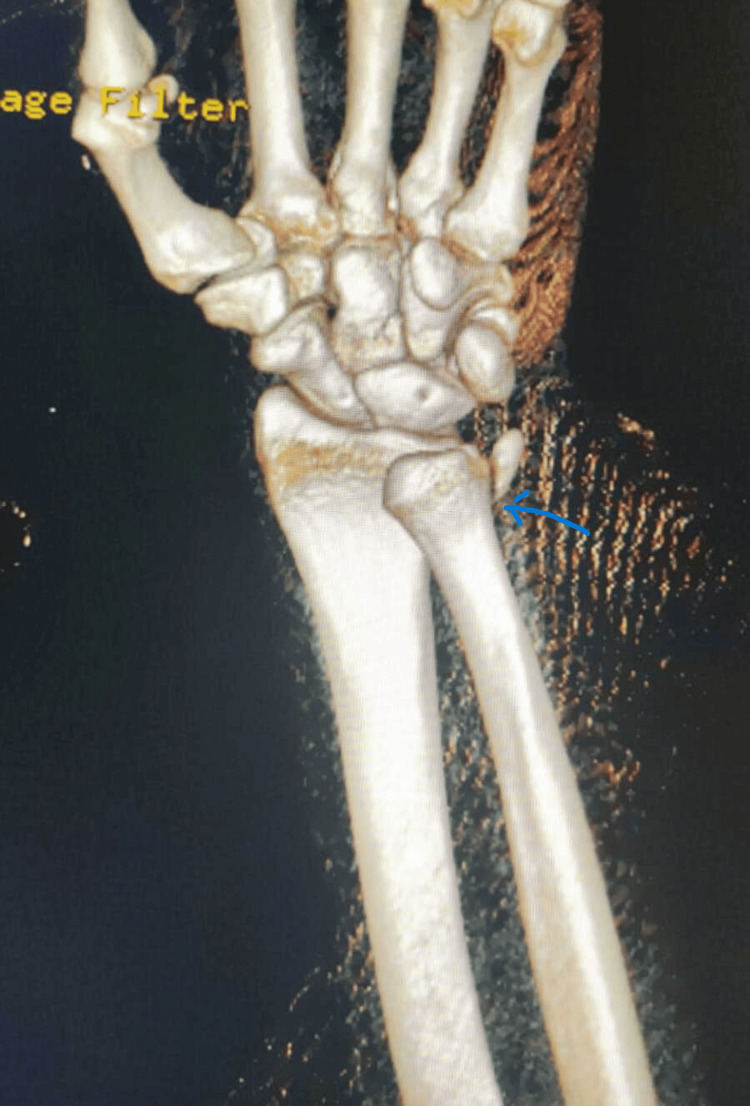
3D computed tomography Computed tomography 3D reconstruction clearly showing volar distal radioulnar joint dislocation associated with an ulnar styloid avulsion fracture (from another view). Blue arrow pointing to the ulnar styloid avulsion fracture with the head going to the volar surface of the radius. Figure created using Philips IntelliSpace PACS (formerly known as iSite) (Amsterdam, Netherlands).

Because of the inadequate radiographs and persistent clinical suspicion, a CT scan was performed. A 3D reconstruction (Figure 3) confirmed volar dislocation of the DRUJ. A second CT 3D image (Figure 4) clearly demonstrated an associated ulnar styloid avulsion fracture.

Closed reduction was performed under conscious sedation using longitudinal traction and directed dorsal‑ulnar pressure on the volarly displaced ulnar head. A distinct "clunk" indicated successful reduction. Post‑reduction examination confirmed restored stability through passive pronation and supination, with intact distal neurovascular status.

**Figure 5:**
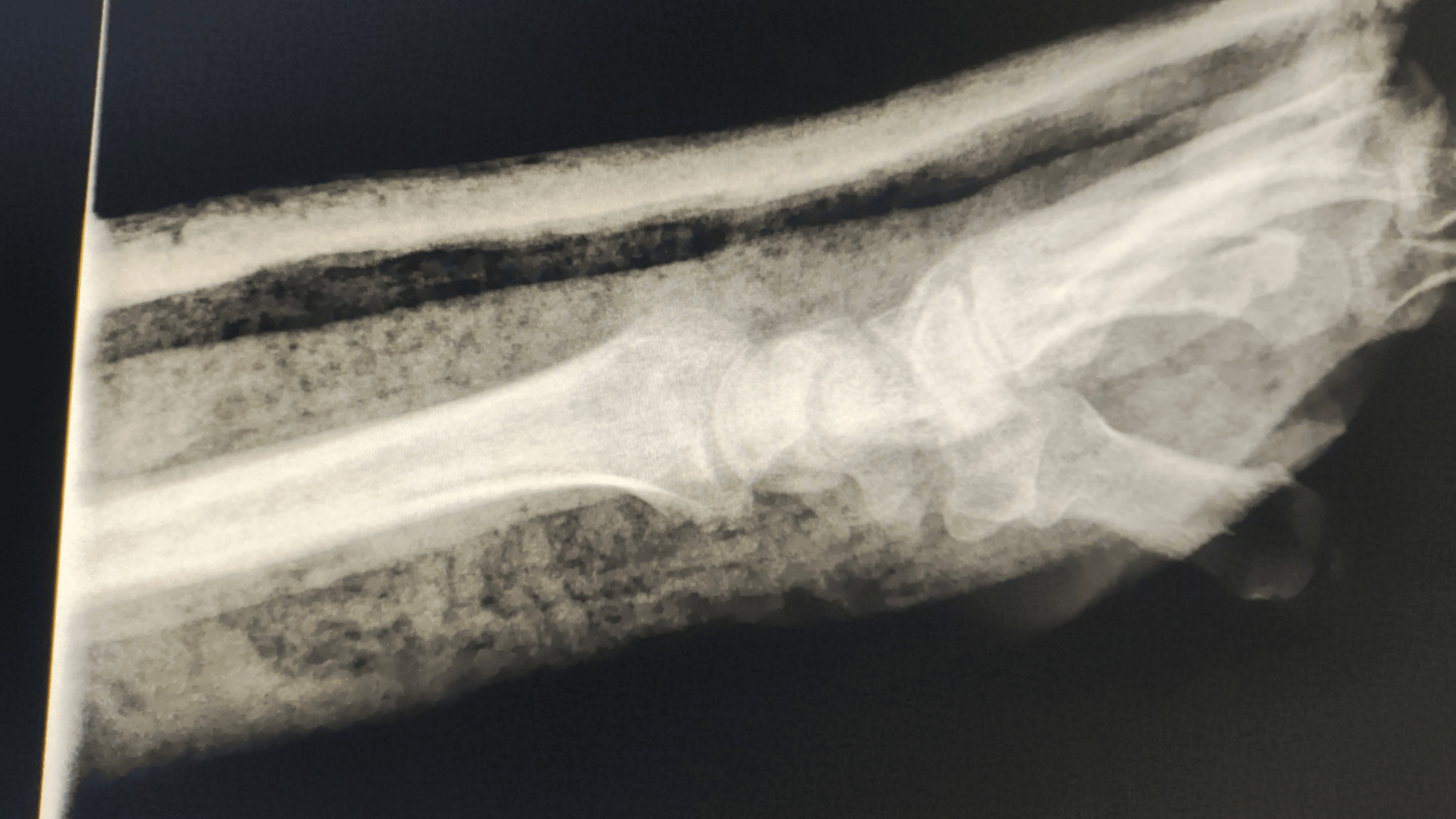
Post-reduction X-ray Post‑reduction lateral wrist radiograph confirming the anatomic reduction of the distal radioulnar joint.

**Figure 6:**
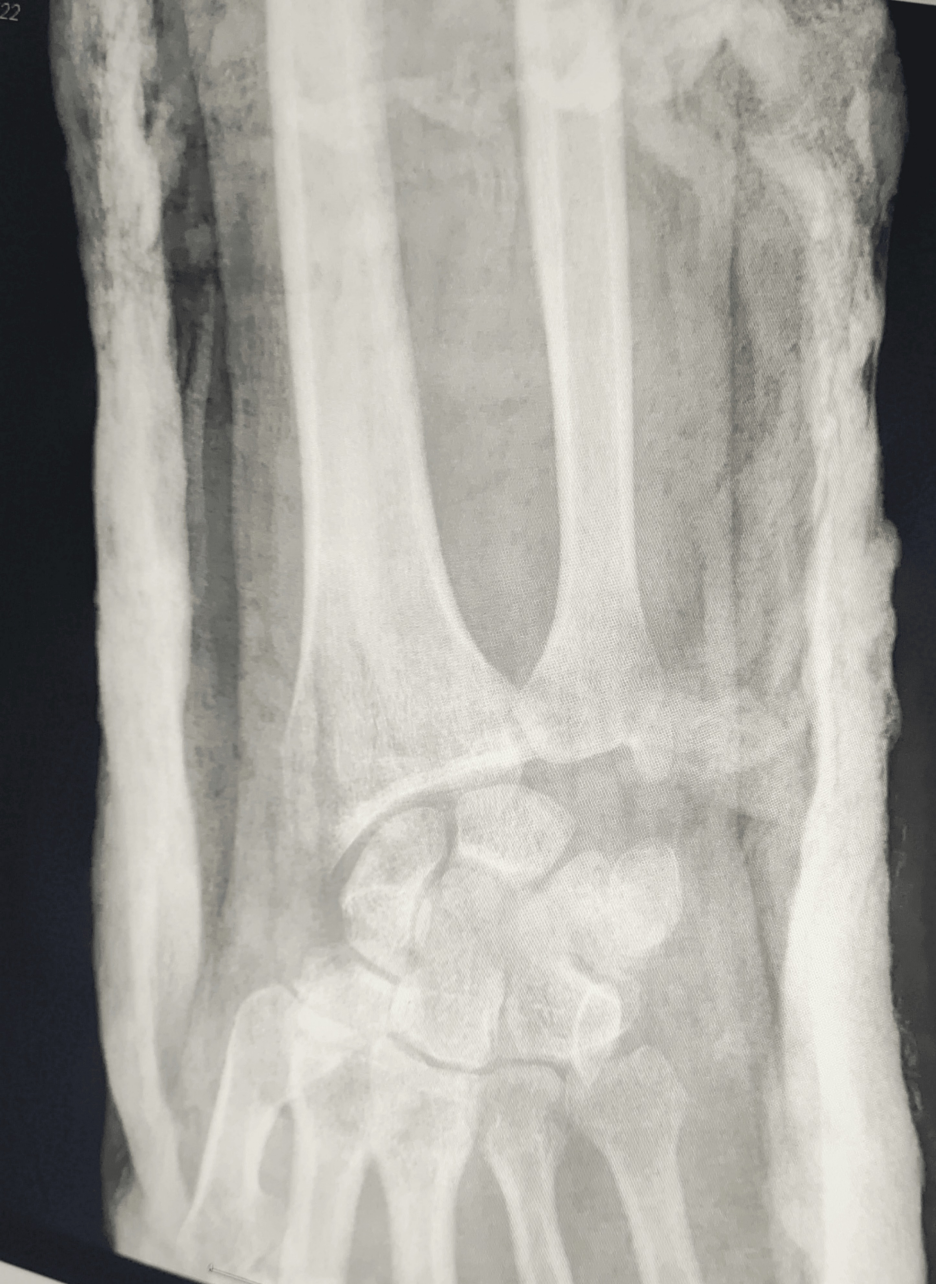
Post‑reduction anteroposterior X-ray Post‑reduction anteroposterior wrist radiograph demonstrating proper alignment of the nondisplaced ulnar styloid fracture.

**Figure 7:**
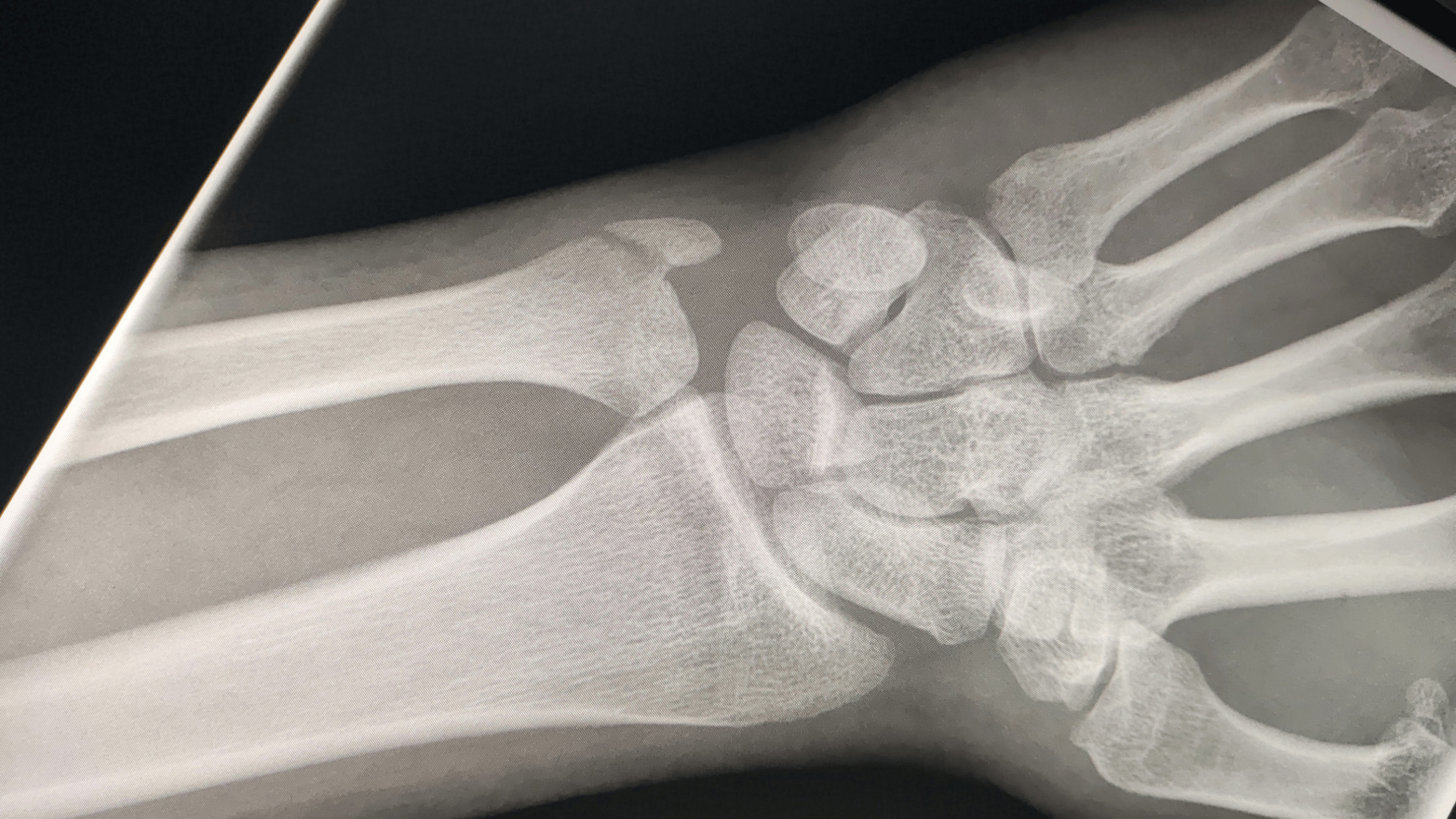
Follow‑up wrist X-ray Follow‑up wrist radiograph without cast showing maintained reduction and stable alignment of the distal radioulnar joint.

A long‑arm back slab was applied. Post‑reduction radiographs confirmed anatomic reduction of the DRUJ and proper alignment of the nondisplaced ulnar styloid fracture (Figures 5-6). Weekly follow‑up during the first three weeks showed maintained reduction and full finger motion. At six weeks, the cast was removed, revealing minimal swelling and mild limitation of wrist motion. Home‑based exercises were initiated. By 11 weeks, the patient achieved full, pain‑free wrist motion comparable to the contralateral side (Figure 7).

## Discussion

Volar DRUJ dislocation is a rare injury, and its combination with an ulnar styloid avulsion fracture is even less frequently reported [1,3]. Diagnostic difficulty often arises from subtle clinical findings and challenges in obtaining proper radiographs, as demonstrated in this case.

When standard radiographs are inconclusive, CT imaging becomes essential for confirming the diagnosis and identifying associated fractures [4]. Recent case reports continue to emphasize the importance of CT and 3D reconstruction in confirming volar DRUJ dislocation [5].

Management strategies vary depending on joint stability after reduction and the presence of associated injuries. Surgical intervention, such as open reduction, ligament repair, or temporary K‑wire fixation, is typically reserved for irreducible dislocations or delayed presentations [2,7]. Several recent reports describe irreducible volar DRUJ dislocations requiring open reduction due to soft tissue interposition [7].

However, other contemporary reports describe successful outcomes with conservative treatment when reduction is stable and no major soft tissue entrapment is present [6]. Our case aligns with this approach.

Home‑based rehabilitation

After cast removal, the patient was advised to follow a structured home‑based rehabilitation protocol consisting of warm fomentation to improve soft tissue elasticity, frequent wrist and finger mobility exercises, and progressive active movements within the pain‑free range. The patient was encouraged to perform these exercises multiple times daily to minimize stiffness and facilitate the recovery of pronation-supination and wrist function. This simple home‑based program contributed significantly to the restoration of full motion by the final follow‑up.

Complications

Reported complications include the following: chronic DRUJ instability, restricted pronation-supination, persistent ulnar‑sided wrist pain, TFCC‑related symptoms, and degenerative changes in chronic or missed cases.

Differential diagnoses

Differential diagnoses include the following: distal radius fracture with apparent DRUJ malalignment, TFCC tear without frank dislocation, Essex‑Lopresti injury, radiocarpal dislocation, and isolated ulnar styloid fracture without DRUJ instability.

Novelty of this case

This report adds value to the limited contemporary literature by presenting the following: a rare combination of volar DRUJ dislocation with ulnar styloid avulsion fracture, high‑quality 3D CT imaging clearly demonstrating the injury pattern, successful conservative management despite the associated fracture, and a clear illustration of how inadequate radiographs may obscure the diagnosis. These points are consistent with emerging evidence from recent reports [4,6].

## Conclusions

Early identification of volar DRUJ dislocation is essential to prevent complications, particularly when associated with an ulnar styloid fracture. Because pain and limited motion may hinder proper radiographic positioning, CT imaging plays a key role in confirming the diagnosis. In this case, prompt evaluation, successful closed reduction, and appropriate immobilization resulted in full functional recovery without surgical intervention. Maintaining a high index of suspicion and ensuring adequate imaging are critical for optimal outcomes.
